# *Salmonella* Bacteriophage Diversity According to Most Prevalent *Salmonella* Serovars in Layer and Broiler Poultry Farms from Eastern Spain

**DOI:** 10.3390/ani10091456

**Published:** 2020-08-19

**Authors:** Sandra Sevilla-Navarro, Pablo Catalá-Gregori, Clara Marin

**Affiliations:** 1 Centro de Calidad Avícola y Alimentación Animal de la Comunidad Valenciana (CECAV), Calle Nules 16, 12539 Castellón, Spain; s.sevilla@cecav.org; 2 Departamento de Producción y Sanidad Animal, Salud Pública Veterinaria y Ciencia y Tecnología de los Alimentos, Instituto de Ciencias Biomédicas, Facultad de Veterinaria, Universidad Cardenal Herrera-CEU, CEU Universities, Avenida Seminario s/n, 46113 Moncada, Spain; clara.marin@uchceu.es

**Keywords:** *Salmonella*, bacteriophages, prevalence, broilers, layers

## Abstract

**Simple Summary:**

There is a lack of knowledge about the impact that phages present in the environment may have against certain *Salmonella* serovars. Thus, an improved understanding of *Salmonella* phage diversity will provide a better insight into the role of phages in *Salmonella* ecology and diversity. The results of this study showed that the poultry farm environment could represent a valuable source of *Salmonella* phages, which are more varied in broiler than in layer farms.

**Abstract:**

The exploration of novel nonantibiotic interventions in the field, such as the use of bacteriophages, is necessary to avoid the presence of *Salmonella*. Bacteriophages are a group of viruses widely distributed in nature, strictly associated with the prokaryotic cell. Researchers have demonstrated the success of phage therapy in reducing *Salmonella* counts in poultry products. However, the impact that phage concentration in the environment may have against certain *Salmonella* serovars is not well understood. Therefore, the aim of this study was to assess *Salmonella* phage prevalence in commercial poultry farms in terms of the production type: layers or broilers. The most prevalent *Salmonella* serovars isolated in poultry production were used for phage isolation. *Salmonella* specific phages were isolated from 141 layer and broiler farms located in the Valencia region during 2019. Analysis of the samples revealed that 100% presented *Salmonella* phages, the most prevalent being against serovar *S.* Enteritidis (93%), followed by *S.* Virchow (59%), *S.* Typhimurium (55%), *S.* Infantis (52%) and *S.* Ohio (51%). These results indicate that poultry farms could represent an important source of *Salmonella* phages. Nevertheless, further studies are needed to assess the epidemiology of phages against other serovars present in other countries and their diversity from the point of view of molecular studies.

## 1. Introduction

*Salmonella* spp. remain one of the main bacteria involved in food-borne outbreaks and are a major public health hazard worldwide [1]. It is estimated that nontyphoidal *Salmonella* worldwide cause around 94 million cases of illness and 155,000 deaths per year [2]. The latest data published by the European Food Safety Authority (EFSA) reported 91,857 human cases, 43.2% of which included hospitalization [3].

There are numerous sources of human salmonellosis infection, but eggs and poultry meat are reported to be the most common sources [3]. The latest data recorded in 2019 showed that 4% of tested flocks were positive for *Salmonella* detection, from which 1.1% were *S.* Enteritidis and *S.* Typhimurium target serovars [3]. However, among those outside the target serovars, the most common reported was *S.* Infantis. Considering the production chain for meat and meat products, the highest percentages of positive samples were found for fresh broilers meat, with *S.* Enteritidis, *S.* Typhimurium, and the *S.a* monophasic Typhimurium variant [3] as the main serovars involved in human outbreaks. In this line, the introduction of National *Salmonella* Control Programmes (NSCP) to control the bacterium at the field level resulted in an important reduction in the prevalence of poultry *Salmonella* serovars in Europe [4]. However, total elimination of the bacterium from poultry flocks is still difficult, and new cases of salmonellosis emerge every year, resulting in economically significant losses for the poultry sector [3].

In addition, the emergence of several *Salmonella* serovars resistant to multiple antibiotics in poultry-derived products underscores a significant food safety and poultry production hazard [5]. For this reason, the exploration of novel nonantibiotic interventions in the field should be studied to avoid the presence of antibiotic-resistant strains [5].

Bacteriophages or phages are a group of viruses widely distributed in nature, whose life cycle is strictly associated with the prokaryotic cell [6,7]. The use of host-specific phages has been promoted as a cost-effective and adaptable approach to control zoonotic bacteria [8,9,10,11]. Moreover, phages seem to be a good alternative due to their self-perpetuating, self-limiting and specificity characteristics [12]. Researchers have demonstrated the success of phage therapy in poultry products, reducing *Salmonella* counts from broiler carcasses after phage administration. Higgins et al. (2005) reduced *Salmonella* counts in 100% of broiler carcasses where phages were inoculated [13]. Moreover, Kang et al. (2013) decreased *Salmonella* counts on chicken skin by up to 3 logs after the application of a single phage [14]. Other research showed *Salmonella* decreasing counts by 1 log on fresh egg shells after application of the phage [7].

However, the impact that phages present in the environment may have against certain *Salmonella* serovars with relevance in food safety is not well understood. Thus, an improved understanding of *Salmonella* phage diversity may provide better insights into the role of phages in *Salmonella* ecology and diversity and facilitate an improved approach toward biocontrol and diagnostics [15,16].

The aim of this study was, therefore, to assess *Salmonella* phage diversity in commercial layer and broiler poultry farms in relation to the most prevalent serovars in the poultry production system in Eastern Spain. Thus, in this study we tested whether occurrence of phages against *Salmonella* was related to the poultry production type.

## 2. Materials and Methods

### 2.1. Salmonella Strain Selection for Phage Isolation

*Salmonella* strains used for phage detection were field strains selected from the strain collection repository from the *Centro de Calidad Avícola y Alimentación Animal de la Comunidad Valenciana* (CECAV), which is the benchmark laboratory for *Salmonella* field strains isolation from poultry farms throughout Spain. The origin of the field strains was the NSCP [4], and each selected strain used in this study was isolated from poultry farms. All selected serovars were those most prevalent in poultry production in Spain [3]: *S.* Enteritidis, *S.* Typhimurium, *S.* Typhimurium monophasic variant, *S.* Kentucky, *S.* Hadar, *S.* Senftenberg, *S.* Ohio, *S.* Infantis and *S.* Virchow. The strains were thawed and revived on nutrient agar (Oxoid Ltd., England, UK) and incubated at 37.5 ± 2 °C for 18 ± 4 h. For characterization of the strains, the antimicrobial susceptibility pattern was performed. To this end, *Salmonella* sensititre plates (Gram Negative MIC Plate) were used to assess antimicrobial susceptibility of isolated strains. A 10 µL aliquot of the inoculum was aseptically transferred to 10 mL sensititre cation-adjusted Mueller-Hinton broth, and plaques were inoculated according to manufacturer instructions. Plates were read at 18 h to 24 h manually by visualization of a growth button on the bottom of the microtitre well using a light box. Reading the results was performed according to the manufacturer’s instructions. The antibiotics selected were those set forth in Decision 2013/653 [17], including: 2 quinolones: ciprofloxacin (CIP, 0.015–8 µg/mL) and nalidixic Acid (NAL, 4–128 µg/mL); 2 B-lactams: meropenem (MERO, 0.03–16 µg/mL) and ampicillin (AMP, 1–64 µg/mL), one phenicol: chloramphenicol (C, 8–128 µg/mL); one pyrimidine: trimethoprim (TM, µg/mL); one tetracycline: tetracycline (TET, µg/mL); one macrolide: azithromycin (AZM, 2–64 µg/mL); one glycylcycline: tigecycline (TGC, 0.25–8 µg/mL); 2 cephalosporin: ceftazidime (CAZ, 0.5–8 µg/mL) and cefotaxime (CTX, 0.25–4 µg/mL); one polymyxin: colistin (COL, 1–16 µg/mL); one potentiated sulfonamide: sulfamethoxazole (SMX, 8–1024 µg/mL), and one aminoglycoside: gentamicin (GN, 0.5–32 µg/mL). Multidrug resistance (MDR) was defined as acquired resistance to at least one agent in three or more antimicrobial classes [18].

### 2.2. Study Sample

A total of 141 poultry farms located in the Eastern Spain were sampled: 108 layer farms (from 41 to 64 weeks of rearing) and 33 broiler farms (ranging from 35 to 42 days of rearing), all of them belonging to three of the main companies in Spain that handle the majority of the broilers and layers reared in Spain (one company from broiler and two companies from laying hens). Farms selected for the study were conventional commercial poultry farms of broilers and layers. All flocks of laying hens analyzed (lines Lohmann and Hyline) were vaccinated against *Salmonella* according to the standard vaccination guidelines. To this end, *Salmonella* vaccination was performed with the vaccine Salmovac 440, a live vaccine given orally in a triple dose through water (day 1, week 6 and week 15) to protect against *S.* Enteritidis and *S.* Typhimurium serovars according to mandatory regulations in the Valencia region [19]. Moreover, layers were reared in cages with a density of 750 cm^2^/hen. With respect to broiler production, all analyzed flocks (lines Cobb and Ross) were reared on the floor in cages containing wood shavings to a depth around 10 cm, and with a density of 33 kg/m^2^. All the animals were kept indoors under controlled conditions equipped with programmable electrical lights, automated electric heating and forced ventilation [20].

### 2.3. Faeces Samples Collection

From each farm, two faeces samples of 150 g were taken from different points of the facility [4]. Once in the laboratory, faeces samples collected from each farm were pooled and placed in sterile pots: 25 g to assess *Salmonella* status of the farm and 10 g for phage detection (as described below).

### 2.4. Salmonella Isolation

Samples were analyzed according to the ISO 6579-1:2017 [21]. Firstly, faeces samples were pre-enriched 1:10 (*v*/*v*) in buffered peptone water 2.5% (BPW, Scharlau^®^, Barcelona, Spain) and incubated at 37 ± 1 °C for 18 ± 2 h. After incubation, the pre-enriched samples were transferred onto a Semi-Solid Modification Rappaport Vassiliadis agar plate (MSRV, Difco^®^, Valencia, Spain), and incubated at 41.5 ± 1 °C for 24–48 h. The resulting culture was used to streak xylose–lysine–deoxycholate (XLD, Liofilchem, Valencia, Spain) and ASAP (ASAP chromogenic media, bioMérieux, Madrid, Spain) agar plates, and incubated at 37 ± 1 °C for 24 h. Next, five typical colonies were streaked onto predried nutrient agar plates (Scharlab^®^, Barcelona, Spain) at 37 ± 1 °C for 24 ± 3 h and confirmed as *Salmonella* spp. using the API (API-20^®^, bioMérieux, Madrid, Spain) biochemical test.

### 2.5. Salmonella Phage Isolation

Phages were isolated from faeces collected by an enrichment procedure [22]. To do so, 10 g of each faeces sample were diluted in 90 mL of Luria Bertani (LB) (VWR Chemicals, Barcelona, Spain) and incubated along with each selected *Salmonella* serovar overnight at 37 °C. After incubation, 2 mL of this enrichment culture was centrifuged 16,000× *g* for 5 min. The supernatant was then filtered through a 0.22 µm membrane.

Phages were isolated and purified in a spot test by the double agar method. Briefly, bacterial suspensions of each serovar were adjusted to an optical density at 600 nm (OD = 600) of 0.2 (~10^8^ CFU/mL) in LB and incubated at 37 °C for 4 h. Then, 200 µL of cultures were added to 5 mL of LB agar (LB with 0.6% agar) tempered to 45 °C and poured onto previously prepared and dried LB basal agar (with 1.6% agar). Then, 10 uL of each filtrate were spotted onto the surfaces of *Salmonella* lawns and incubated overnight at 37 °C. After the incubation, morphologically different plaques were selected and resuspended in 1 mL of PBS. Ten-fold serial dilutions of the phage suspension were plated by the double agar layer method, and phages that produced clear plaques were selected. This procedure was repeated three times to obtain a single type of phage [23].

### 2.6. Statistical Analysis

We tested whether occurrence of phages against *Salmonella* was related to the poultry production system. To do so, we fitted a generalized linear model (GLM) where occurrence of *Salmonella* phage was the response variable and the sample type (faeces from different broiler and layer farms), *Salmonella* serovar (n = 9), genetic lines (n = 2, for both poultry production type), poultry companies (n = 1 and n = 2, for broilers and layers, respectively), husbandry (n = 1), *Salmonella* vaccine strain (n = 1) were the factors.

For this analysis, the error was designated as having a binomial distribution and the probit link function was used. Binomial data for each sample were assigned a 1 if a *Salmonella* phage was isolated or a 0 if not. A *p*-value < 0.05 was considered to indicate a statistically significant difference. Differences in binomial traits for variables, genetic lines, poultry companies, husbandry and *Salmonella* vaccine strain, were not significant and were excluded from the model. Finally, a descriptive analysis of the patterns obtained against different *Salmonella* serovars per farm, and antimicrobial resistance of the strains, was carried out. Analyses were carried out using a commercially available software program (SPSS 21.0 software package; SPSS Inc., Chicago, IL, USA, 2002).

## 3. Results

**Figure 1:**
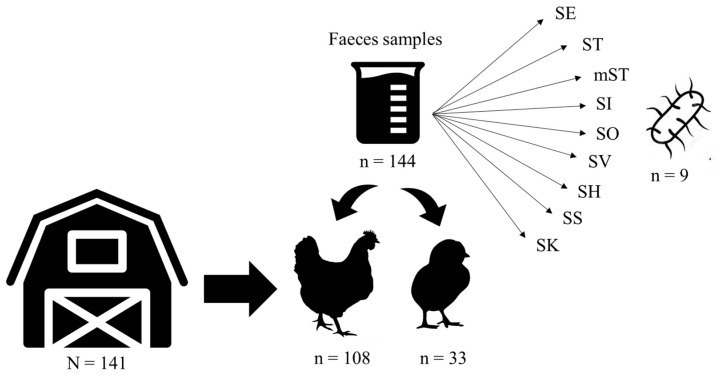
Diagram of the experiment carried out to assess the specific phage isolation in each farm (n = 141) per each *Salmonella* serovar (n = 9). SE: *S.* Enteritidis; ST: *S.* Typhimurium; mST: *S.* Typhimurium monophasic variant, SK: *S.* Kentucky; SH: *S.* Hadar; SS: *S.* Senftenberg; SO: *S.* Ohio; SI: *S.* Infantis; SV: *S.* Virchow.

In this study, a total of 141 faeces samples were collected from poultry farms. From each, 141 pools of 25 g were analyzed to assess *Salmonella* status of the farm, and 1269 analyses were done for specific phages isolation (farm × serovar) (Figure 1). No *Salmonella* was detected in any farm, although *Salmonella* phages were detected in all farms sampled, at least against one of the serovars included in this study.

### 3.1. Salmonella Antimicrobial Susceptibility Characterization

From different *Salmonella* serovars from the poultry sector included in this study (n = 9), 56% were resistant to at least one of the fourteen antibiotics tested, and 44% were MDR to 3 or more of the groups of antibiotics tested. *Salmonella* serovars MDR were *S.* Typhimurium monophasic variant, *S.* Typhimurium and *S.* Virchow. The highest percentages of antimicrobial resistance (AMR) were found to be TET (44%) followed by AMP (33%), NAL (33%), SMX (22%) TMP (11%), and CHL (11%). Resistance to MERO, AZM, TGC, CAZ, COL, GN and CTX was not observed.

### 3.2. Salmonella Phage Prevalence in Poultry Farms

**Figure 2:**
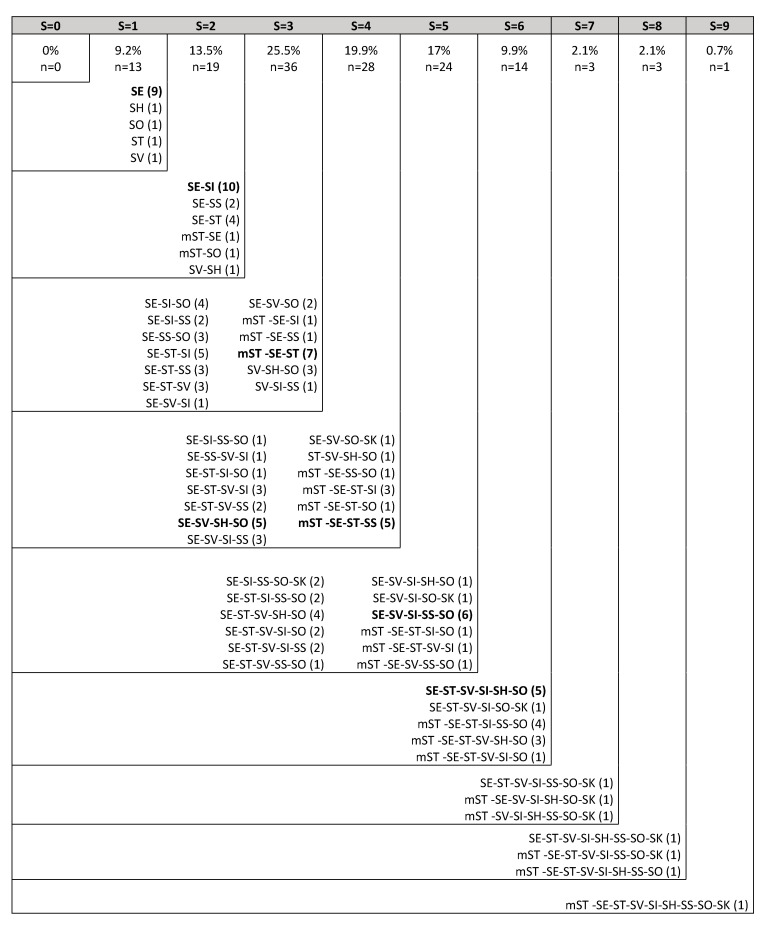
Phage lysis spectrum patterns obtained against different *Salmonella* serovars per farm. S: number of *Salmonella* serovars sensitive against phages per farm; n: Number of farms; SE: *S.* Enteritidis; ST: *S.* Typhimurium; mST: *S.* Typhimurium monophasic variant, SK: *S.* Kentucky; SH: *S.* Hadar; SS: *S.* Senftenberg; SO: *S.* Ohio; SI: *S.* Infantis; SV: *S.* Virchow. The number of farms where each phage pattern was obtained is shown within parentheses. Most prevalent patterns are represented in bold letters.

From 1269 analyses done for specific phages isolation (farm × serovar), statistically significant differences were found according to poultry production type (*p* < 0.05). Layer and broiler farms presented at least one *Salmonella* serovar-specific phage in 42% (408/972) and 53% (156/297) of faeces samples analyzed, respectively. From farms analyzed, 9.2% (13/141) of samples presented phages against one serovar, 13.5% (19/141) against two serovars, 25.5% (36/141) against three serovars, 19.9% (28/141) against four serovars, 17% (24/141) against five serovars, 9.9% (14/141) against six serovars, 2.1% (3/141) against seven serovars and eight serovars, and 0.7% (1/141) against all serovars. The lysis spectrum patterns are described in Figure 2.

### 3.3. Prevalence of Salmonella Phages per Serovar and Poultry Production Type

Regardless of the poultry production type (layers or broilers), statistically significant differences were shown among serovar-specific phages isolated (*p* < 0.05). The most prevalent *Salmonella* phage present was against *S.* Enteritidis serovar (93%), followed by *S.* Virchow (59%), *S.* Typhimurium (55%), *S.* Infantis (52%) and *S.* Ohio (51%) (Table 1).

With respect to layers, statistically significant differences were shown among *Salmonella* phages isolated (*p* < 0.05). The highest percentage of phage present was against *S.* Enteritidis (94%), followed by *S.* Typhimurium (53%), *S.* Infantis (52%), *S.* Virchow (47%) and *S.* Ohio (44%). In addition, regarding broiler production, statistically significant differences were shown among *Salmonella* phages isolated (*p* < 0.05). The highest percentage of phages was against *S.* Virchow (97%) and *S.* Enteritidis (91%), followed by *S.* Ohio (76%) and *S.* Typhimurium (64%). However, none of the broiler samples collected presented phages against *S.* Kentucky serovar (Table 2).

**Figure 3:**
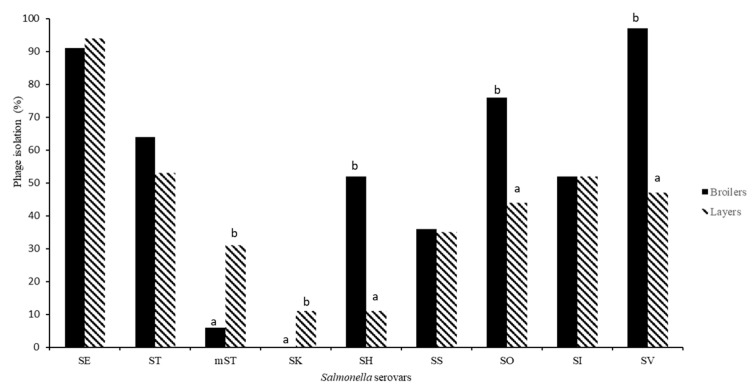
Percentage of *Salmonella* phages isolated related to serovars and poultry production type (layers vs. broilers). ^a, b^ Superscript indicates significant differences in *Salmonella* phage isolated according to poultry production type. SE: *S.* Enteritidis; ST: *S.* Typhimurium; mST: *S.* Typhimurium monophasic variant, SK: *S.* Kentucky; SH: *S.* Hadar; SS: *S.* Senftenberg; SO: *S.* Ohio; SI: *S.* Infantis; SV: *S.* Virchow.

Moreover, statistically significant differences were shown between different poultry production type and phages isolated. From broiler farms, a higher prevalence of phages was observed against *S.* Virchow, *S.* Ohio and *S.* Hadar. Conversely, the highest phage prevalence against the monophasic *S.* Typhimurium variant and *S.* Kentucky, was obtained from samples from laying hens (*p* < 0.05). No statistically significant differences were found between poultry production type, and phage isolation against *S.* Enteritidis, *S.* Typhimurium, *S.* Infantis and *S.* Senftenberg strains (*p* > 0.05) (Figure 3).

## 4. Discussion

The diversity of *Salmonella* phages in poultry farms regarding their production type (broilers or layers) and the most prevalent *Salmonella* serovars in the Eastern Spain were analyzed in this study. Although *Salmonella* spp. were not present in any of the farms assessed, phages from several serovars of public health and poultry production importance were present in 100% of the samples collected. These results showed that although the bacterium is not present in the farm environment, its specific phages can remain in it.

It is claimed that AMR will be the main cause of deaths worldwide by 2050, overtaking other major causes of deaths such as cancer or road traffic accidents [24,25]. For this reason, the reduction of antimicrobial use at the field level throughout Europe is one of the most important aims in the poultry sector [26]. Results of this study showed *Salmonella* strains with a high percentage of antimicrobial resistance, especially against TET, AMP and NAL, three of the antibiotics most frequently used to treat poultry, and also used against human diseases [27,28]. Although *Salmonella* treatment with antibiotics is banned in the EU, its resistance to antibiotics could be acquired from different sources, such as the environment or antibiotics used to control other infections (*E. coli*) [29]. Phage patterns obtained against different *Salmonella* serovars per farm in this study indicated that the environment of animal farms, especially poultry operations, could represent an important source of *Salmonella* phages against several serovars [30,31]. In this sense, the phages obtained could be effective to combat these antibiotic-resistant strains, with the aim of controlling *Salmonella* AMR and its spread to the food chain [32].

Regarding *Salmonella* phages per serovar and poultry production type, *S.* Enteritidis, *S.* Typhimurium and *S.* Typhimurium monophasic variant phages were three of the phages most frequently isolated in poultry farms. This could be explained by the strict vaccination programs implemented in the poultry production system. Vaccination against *S.* Enteritidis is mandatory in all commercial layer flocks, and optional for layer and broiler breeders [33]. In addition, the vaccination programme is stricter in the Valencia region where, since 2008, it is mandatory to vaccinate not only against *S.* Enteritidis, but also against *S.* Typhimurium [19]. Live vaccination in poultry maintains the *Salmonella* vaccine strain in birds, as well as the house environment [34,35,36], and could encourage phage presence in the field. In this context, the latest data recovered from official checks in the Valencia region showed that 100% of *S.* Enteritidis strains isolated from rearing layers were *S.* Enteritidis vaccine strains (unpublished data). Moreover, specific phages against *S.* Typhimurium monophasic variant have been found, which may be explained by the mandatory oral administration of *S.* Typhimurium vaccine, which could provide cross-immunization against *S.* Typhimurium monophasic variant [37].

A high prevalence of phages against *S.* Ohio, *S.* Infantis and *S.* Virchow have been found in this study; these are three of the main serovars isolated in the Valencia region from the NSCP (unpublished data). These results are in line with other researchers, who stated that the presence of phages in the farm environment would suggest the bacterial strain has been present at some point in the recent past [12,38]. In addition, this fact could be used for the indirect detection of pathogens based on their specificity towards bacteria [32,39]. In this line, phages against *S.* Virchow, *S.* Hadar and *S.* Ohio were observed to be more prevalent in broilers than in layers. These results are in accordance with data recovered from the *Salmonella* control programme in the Valencia region, as neither *S.* Virchow nor *S.* Hadar were isolated from laying farms (unpublished data). Moreover, Marin and Lainez (2009) also demonstrated that the main serovars isolated from broiler farms in the Valencia Region were *S.* Virchow, *S.* Ohio and *S.* Hadar [40]. On the other hand, no statistical differences have been found between the poultry production type and the presence of phages against *S.* Enteritidis, *S.* Typhimurium, *S.* Infantis, and *S.* Senftenberg. This result could be related to the historically close relationship between these serotypes and both layer and broiler production systems [41].

## 5. Conclusions

In conclusion, the results of this study showed that the poultry farm environment could represent a valuable source of *Salmonella* phages. A wide *Salmonella* phage diversity was present in the broiler and layer farms analyzed, being more varied in broilers. Nevertheless, further studies are needed to study the epidemiology of phages against other serovars present in other countries and its diversity from the point of view of molecular studies.

## Figures and Tables

**Table 1:** Percentage of *Salmonella* phages isolated from poultry farms related to *Salmonella* serovars included in the study.

Strain	n	%	SEM
SE	131	93 ^g^	0.022
SV	83	59 ^f^	0.041
ST	78	55 ^f^	0.042
SI	73	52 ^f^	0.042
SO	72	51 ^f^	0.042
SS	50	35 ^d,e^	0.040
mST	36	26 ^c,d^	0.037
SH	29	21 ^b^	0.034
SK	12	9 ^a^	0.023

^a, b, c, d, e, f, g^: Percentage with different superscripts means statistically significant difference within column; SEM: Standard error of the mean; n: Number of positive farms; SE: *S.* Enteritidis; ST: *S.* Typhimurium; mST: *S.* Typhimurium monophasic variant, SK: *S.* Kentucky; SH: *S.* Hadar; SS: *S.* Senftenberg; SO: *S.* Ohio; SI: *S.* Infantis; SV: *S.* Virchow.

**Table 2:** Percentage of *Salmonella* phages isolated per serovar within poultry production type.

	Poultry Production Type					
	Layers			Broilers		
Strain	n	(%)	SEM	n	(%)	SEM
SE	101	94 ^E^	0.024	30	91 ^e,f^	0.050
ST	57	53 ^D^	0.048	21	64 ^d,e^	0.084
mST	34	31 ^B^	0.045	2	6 ^b^	0.042
SK	12	11 ^A^	0.030	0	0 ^a^	0.000
SH	12	11 ^A^	0.030	17	52 ^c,d^	0.087
SS	38	35 ^B,C^	0.046	12	36 ^c^	0.084
SO	47	44 ^C,D^	0.048	25	76 ^e^	0.075
SI	56	52 ^D^	0.048	17	52 ^c,d^	0.087
SV	51	47 ^D^	0.048	32	97 ^f^	0.030

^a, b, c, d, e, f^: percentage with different superscripts means statistically significant difference within column; ^A, B, C, D, E^: percentage with different superscripts means statistically significant difference within column; SEM: Standard error of the mean; n: number of samples positive for the presence of a phage against *Salmonella*; SE: *S.* Enteritidis; ST: *S.* Typhimurium; mST: *S.* Typhimurium monophasic variant, SK: *S.* Kentucky; SH: *S.* Hadar; SS: *S.* Senftenberg; SO: *S.* Ohio; SI: *S.* Infantis; SV: *S.* Virchow.

## Abbreviations

The following abbreviations are used in this manuscript:

EFSA	European Food Safety Authority
AMR	Antimicrobial Resistance
NSCP	National *Salmonella* Control Programmes
CIP	Ciprofloxacin
NAL	Nalidixic Acid
MERO	Meropenem
AMP	Ampicillin
C	Chloramphenicol
TM	Trimethoprim
TET	Tetracycline
AZM	Azithromycin
TGC	Tetracycline
CAZ	Ceftazidime
CTX	Cefotaxime
COL	Colistin
SMX	Sulfamethoxazole
GN	Gentamicin
MDR	Multidrug resistance
LB	Luria-Bertani
OD	Optical density
GLM	Generalized Linear Model
